# Belonging and ‘Unbelonging’: Jewish refugee and survivor women in 1950s Britain

**DOI:** 10.1080/09612025.2015.1123028

**Published:** 2016-04-11

**Authors:** Angela Davis

**Affiliations:** ^a^ Department of History, University of Warwick, CoventryCV4 7AL, UK

## Abstract

This article analyses the life stories of female Jewish refugees and survivors in 1950s Britain in order to explore their relationship with the existing Jewish community and wider society. The paper is based on an analysis of twenty-one oral history testimonies from the Jewish survivors of the Holocaust collection held at the British library. Around 50,000 Jewish refugees from Central Europe came to Britain in the 1930s after fleeing from Hitler. In addition, a relatively small number of camp survivors and former hidden children settled in the country after the war; the Board of Deputies of British Jews Demographic Unit estimates the figure at 2000. This article considers how these refugee and survivor women tried to find a place for themselves within 1950s Britain. Looking at their experiences of arrival, work and home, it reflects upon the discrimination and hostility they faced, and they ways they tried to deal with this. Finally it discusses what this meant for their sense of belonging or ‘unbelonging’.

## Introduction

In *Imaging Home*, in which she focused on the experience of non-Jewish women migrant workers from Europe and the former colonies, Wendy Webster reflected that:

Unbelonging is a main theme in many women's accounts of their arrival in Britain as migrant workers or as refugees. The meaning of home is a place from which they are in exile or a place which has been destroyed by war in the loss of family and community.^1^

Separated from their original homes they found they were also denied any place in Britain, a country they had thought would welcome them.^2^ Through analysing oral history interviews with twenty-one Jewish women as part of the British Library's Jewish Survivors of the Holocaust collection, this article explores Webster's notion of belonging and ‘unbelonging’ for Jewish refugee women. The article considers how these women struggled to find a place for themselves within 1950s Britain, both within the Jewish community and wider society. Drawing on themes highlighted by Webster as demonstrating the exclusion of migrant women from constructions of home and nation in 1950s Britain—their accounts of arrival, and experiences of work and home—the article examines attitudes towards Jewish refugee women. Throughout it reflects upon the discrimination and hostility they faced, both as Jewish women and foreigners, but also the ways they tried to deal with this. Finally it discusses what this meant for their sense of identity.

Jewish women have often been disregarded in histories of twentieth-century Britain. In part, this is the result of the marginalisation of Jewish history,^3^ ^4^ but also reflects how the experiences of women have been sidelined within Jewish history.^5^ In recent decades this picture has been changing as gender historians have demonstrated the limitations of focusing on the public lives of a male elite.^6^ However, the existing scholarship largely concentrates on the years between 1870 and 1918. The historiography of British-Jewish women in the post-1920 period remains, in Tony Kushner's words ‘almost non-existent'.^7^ Kushner's own work on Jewish women refugees entry into domestic service in the 1930s and 1940s is one notable exception.^8^ However, the experiences of Jewish women after 1945 have yet to receive serious attention. This silence is important because while Jewish women in mid-century Britain may have represented something less than half of one percent of the population, to quote Kushner, ‘until the experience of these and other such “marginal” women are considered we shall continue to produce incomplete and unsatisfactory histories of Britain'.^9^ This article will therefore place the experiences of Jewish women in the context of indigenous women and other immigrant groups in order to see what was particular about the Jewish experience and what this tells us about 1950s Britain.

## Sources and methodology

This paper has employed interviews from the Jewish survivors of the Holocaust collection held at the British Library. The collection contains interviews from two oral history projects, the Living Memory of the Jewish Community and the Holocaust Survivors' Centre Interviews. Interviews with twenty-one women have been selected for this study. The article is based on analysis of digital copies of the recordings of the interviews which are available to listen to through the British Library website. Where available, transcripts have also been used to supplement these audio recordings.The interviews took place between between 1988 and 1998 with twelve different interviewers. The interviewees chosen here were born between 1898 and 1948 and give a varied sample in terms of social background, geography and religiosity. They included women who migrated to Britain before the Second World War, for economic reasons as well as refugees, and those who came after the war. Eighteen were born outside of the UK, in Austria, Czechoslovakia, France, Germany, Poland and Russia. While all were living in Britain by the 1950s, they had different migration trajectories. These factors shaped their accounts.^10^ Indeed it is important to recognise the multiplicity of experiences within survivors' stories.^11^

In recent years, a number of scholars have considered the benefits and limitations of reusing oral history interviews in research.^12^ While a rich resource, the process also brings challenges in terms of ethics, interpretation and understanding. Peter Jackson, Graham Smith and Sarah Olive have stressed that reusing oral sources ‘requires a rigorous and reflexive process of recontextualisation and a full appreciation of the dialogical nature of life history research'.^13^ However, despite the large number of existing Holocaust survivor testimonies, Kushner argues that ‘the use that is to be made of this material has hardly been subject to debate'.^14^ The reuse of all oral testimony raises ethical dilemmas, but survivor testimonies present particular difficulties. Discussing the use of holocaust testimonies from a social science perspective Rachel Einwohner asks whether subjecting the text of oral testimonies to qualitative analysis ‘detracts from the humanity of the individual survivor’.^15^ Einwohner makes use of Janet Jacobs's concept of ‘double vision’ to characterise these ethical dilemmas. In her article about the ethics surrounding the use of photographic images collected from Holocaust memorials Jacobs described ‘double vision’ as occurring where the researcher ‘is at once both a witness to crimes against humanity and an ethnographic observer in search of qualitative data’.^16^ In order to meet some of these challenges I have gone back to the original recordings in order to gain a greater sense of the dynamic of the interview and understanding of how the women told their life stories. I also believe that by focusing on their whole life stories, and particularly their accounts of how they rebuilt their lives after the holocaust, the article can, to use the terminology of Einwohner, reinforce their humanity, by demonstrating the importance of considering their lives beyond the holocaust.

Moreover, despite the problems in reusing testimonies, using archived interviews provides a way of accessing the subjective experiences of Jewish women living in 1950s Britain that it would be impossible to achieve through employing other sources. The history of Jewish refugee and survivor women as they tried to rebuild their lives in 1950s Britain is currently absent from the historical record.^17^ Reconstructing the lives of these women is a central purpose of this article. They were a marginal group on many levels: a minority within a minority group, women, and refugees. However, while their numbers were small it is important that they should not be written out of British history. Moreover their experiences reveal something about the nature of British society in 1950s Britain. How they were received sheds light on the host nation, its attitudes towards religious and ethnic minorities and its behaviour towards refugees. It shows how the nature of post-war reconstruction meant that their wartime experiences were silenced. In order to find a place in 1950s Britain these women often felt compelled to hide who they were. Studying the lives of Jewish refugee women in 1950s Britain therefore also helps to problematize the black/white binary which often characterised accounts of post-war immigration both contemporaneously and in later accounts.^18^

## Arrivals

Although restrictions on immigration were strictly enforced in Britain during the 1930s,^19^ Louise London estimated that between 1933 and 1939 around 90,000 refugees were allowed to enter Britain, about 85 to 90% of whom were Jewish.^20^ One in three, or 20,000 of the refugees who came to Britain before the war came as domestic servants, and therefore the majority were women.^21^ In 1938 and 1939 the British government, with help from Jewish and Quaker organizations, also rescued 10,000 Jewish and non-Aryan children from Germany, Austria, Poland, and Czechoslovakia.^22^ Immediately after the war a small number of Jewish refugees, who had close relatives in Britain but had been refused entry under the narrow terms of the Home Office's Distressed Relatives scheme, arrived under the Ministry of Labour's foreign domestic worker scheme.^23^ In the later 1940s and 1950s they were joined by 50,000 to 60,000 Central European Jews fleeing communist regimes and 2000 or 3000 Jews who migrated from the former British colonies of India, Iraq and Egypt.^24^

Webster has shown that while ‘Black migrants sometimes made the journey to Britain with optimism’, their ‘accounts of arrival record a sense of shock’ that the England they arrived to did not live to their imagination and that they were not welcome there.^25^ The experience for Jewish refugees was somewhat different. They also recalled that austere, wartime or post-war England was not the glamorous place they had expected. However, fleeing antisemitism on the continent they often expected prejudice and discrimination from wider British society. Instead their accounts centre on the lack of welcome, hostility, and sometimes exploitation they received from within their own family and the British Jewish community, which they had not expected to encounter but often did.

Wlodja Robertson was born in 1931 in Warsaw. She came to England in 1946 to join her father who had got out earlier, after being in hiding during the war. Wlodja travelled to England via Sweden which was ‘terribly exciting. Because … Sweden hasn't been in the war, and there was this city full of electric lights, and food, and oranges I had not seen, and bananas, for years.’ She thought that London would be ‘even more exciting’ but found it was ‘very black'.^26^ Edith Birkin was born in1927 in Prague. After surviving the concentration camps, she went to Northern Ireland in 1946 to join her sister who had got out of Czechoslovkia in 1939. She described it as being ‘Victorian. It was like something a hundred years previously.’ She found it to be ‘very depressing. The weather was dreadful. It was raining all the time, the skies were grey. People were grey.’^27^ After a year at school in Londonderry Edith moved to London, which she also thought was ‘very depressing’. She added, ‘there was rationing and nothing much was happening. And people were very reserved and … they weren't outgoing and warm and fun.’^28^ Even the food was ‘uninteresting’.^29^ Unlike Edith and Wlodja who arrived as teenagers, Margaret Augstein was in her thirties when she came to England in 1948; however, she drew a similar picture:

in those days I was not happy here. It was more austere. Life was more austere in England than it was in Prague. I lived in much worse conditions here than I lived in Prague.^30^

Sometimes the promises made by British relatives did not meet the reality of life there. Barbara Stimler and her friend Fay arrived to England from Poland on the same boat in August 1946. Barbara explained what happened when they arrived at Fay's uncle's house:

that was the East End of London in Cable Street, poor Fay she got white like a sheet, it was really dreadful … Because they were writing to her letters, ‘Oh you will become a princess here’, and all this and poor Fay was really disappointed.^31^

Unsurprisingly, interviewees who had suffered the extremes of Nazism could also be afraid of state antisemitism when arriving in Britain. Barbara Stimler was born in 1927 in Poland. An Auschwitz survivor, she came to England in 1946 to join her grandmother and uncles who were living there. Describing a visit to the Foreign Office shortly after she arrived she recalled her surprise that when her details were taken no-one asked what her religion was, ‘because in Poland everywhere you went you had to tell your religion'.^32^ Ruth Foster was born in 1922 in Lingen, Germany. Ruth, who was also a camp survivor, came to England in 1947 to join her husband who had immigrated to Britain under the 1947 Polish Resettlement Act.^33^ Recalling her arrival she explained:

I was the first German-born woman to enter Great Britain after the war. But the fright was still in my bones … And when I went to collect my documents an English officer with an air-force moustache got up from his seat behind this trestle table, from where we were given our documents, he stood up, he must have been six foot tall, and he said, ‘Mrs Freudenheim’, when he saw my papers. And I thought, now he sends me back. Because the fright from all those years were still in our bones, and in my bones. So he shook me by the hand and he said, ‘Mrs Freudenheim, when you board that ship … you are on UK soil’. So I said, ‘But I want to go to England to join my husband’. I didn't know what he meant.^34^

The stories of arrival narrated by Barbara Stimler and Ruth Foster reveal the complexity of their feelings as they told both of their fear of discrimination by the British state, but also that they also found they had nothing to fear.

Some interviewees talked of being well-received by the British public. Lily Herdan was born in 1908 near Budapest. She arrived in England 1949 to join family there after surviving the Budapest ghetto. Discussing her reception she said:

Well, I was very lucky. I lived in a small place, which was a town, Millom, in Cumberland … and the English, the people in Millom were very nice to us, they heard what we had to go through, and, and helped, they were kind and friendly, and it was, it was really very nice.^35^

However it is interesting that Lily thought she was ‘lucky’ implying she did not think that all refugees had such a positive experience. Renate Collins was born 1933 in Czechoslovakia. She came to England in 1939 as part of the Kindertransport to stay with a non-conformist minister and his wife in South Wales. Her narrative centred on the close relationship she developed with her adoptive parents. Her love for them enabled her to tell a positive story of her forced migration. The couple, ‘treated me as if I was their own … I was certainly made to feel one of the family by my mother's relations, and by father's.’^36^ Indeed the whole community had welcomed her: ‘of course, everybody had been told that this little Czech girl was coming, and would they please be nice to her.’^37^

Anna Smith was not so ‘lucky’. Born in 1932 in Prague she came to England in 1939 to live with a friend her mother had made through the girl guides. Her foster family, who later adopted Anna after the war when it became clear her own family had not survived, were non-conformist Christians living in a south of England seaside resort. Anna was unhappy with the family and thought she was ill-treated by the woman who became her adoptive mother. Interestingly she did not think her adoptive mother's behaviour was due to antisemitism but she did face ‘a lot of antisemitism from my adoptive father’, even though, ‘he thought quite a lot of me actually’.^38^ Claudette Kennedy also encountered antisemitism from within her family. Claudette was born in 1910 in Paris. She survived Auschwitz but her first husband perished. A scientist, Claudette arrived in England in 1950 after marrying her second husband, who was British and not a Jew, after they met at an academic conference. She was not well-received by her new father-in-law who did not want to accept the couple's children:

because of them being Jewish. He thought it was really terrible that the children would never know what they were, Jewish or not Jewish. They would never be accepted anywhere.^39^

Jewish families could be equally unwelcoming, feeling their refugee relatives were ‘poor relations’. Barbara Stimler went to live with her aunt and uncle after the war:

I think that they thought, as civilisation was going forward in England, in Poland it stood still, because my aunty would ask me ‘Oh did you have any cinemas?’ or something like that … Anyway I think that they thought they'd brought a girl from a camp, she can't speak and she can't count and she can't read.^40^

Freda Wineman had a difficult relationship with her in-laws after she came to England to marry her husband in 1950s. She said they were ‘very, very hurtful at times’. They felt she was not good enough for her husband, firstly because she had no money; secondly because she was ‘not religious enough in their eyes’; and, ‘Thirdly, they couldn't understand why other Jews could save themselves, and we hadn't saved ourselves. And I tried to explain that six million Jews didn't save themselves.’^41^

Other interviewees talked of feeling out of place amongst British Jewry. Margaret Gold was born in 1920 near Budapest. She came to England in 1957 with her husband and son after leaving Hungary because of the Uprising. Recalling her arrival she said:

Well, when I came here, I joined immediately, the Jewish community, because I felt terribly lonely. Unfortunately, I joined the wrong community, where I was treated as a foreigner, and nobody cared about me. I was a member for more than two years, and nobody talked to me one single word. So I left them, I was terribly upset about that, they were really nasty to me.^42^

Rena Zabielak was born in Warsaw in 1924. After going into hiding during the war she came to England in 1946 to marry her husband, who was her uncle through marriage, who had family in England. When asked how she got on with the local Jewish community when she arrived and whether they helped her she replied: ‘When I first arrived I didn't have any contact and they were not nice to us … They couldn't care a hoot.’^43^

## Work and home

Dolly Smith Wilson suggests that in the 1950s ‘men and women existed in two separate labour markets, one for men, considered the real workers, the other for women, considered low-paid auxiliaries working on the side, unrelated to their real role as wives and mothers'.^44^ However, Webster has shown how ‘black women were not constructed as economic dependents of men’, and in consequence their ‘main role in the post-war welfare state was to subsidize it through their labour'.^45^ For Jewish women the opposite was true and they were less likely to be economically active than their non-Jewish counterparts. In a 1950 *Jewish Chronicle* survey, which included just under 2000 women, only 11% of Jewish women were economically active, as compared to 34% in the general population.^46^ Kushner argues that the male full employment of the post-war years enabled the middle-class Anglo-Jewish ideal of female domesticity to be achieved on a wide scale.^47^ Discussing interviews conducted with both Jews and non-Jews in the East End of London in the early 1950s, Hannah Neustatter found that, ‘Jewish women are normally expected to confine themselves to household duties’ and ‘Jewish working-class men tend to deprive themselves or luxuries rather than allow their wives to share the wage-earning burden'.^48^ Jewish women were also more likely to get married than their non-Jewish counterparts.^49^

This domesticity of Jewish women reflected the celebration of the home both within Jewish culture, but also within 1950s Britain. Mothers have always been central to the maintenance and reproduction of the Jewish home. In the diaspora mothers both produced and preserved the Jewish people in exile.^50^ Mothers enjoy a culturally bestowed authority with respect to childrearing in Jewish tradition. While women's sphere in Judaism is separate and different from that of men, women's particular contributions to Jewish life are viewed to be in the areas of relationship, family and community.^51^ Within post-war Britain women's main role was also seen too be in the home.^52^ The Birmingham Feminist History Group noted how writing and thinking on women in the 1950s, by feminists and non-feminists alike, tended to take place within a framework which accepted the primacy of the woman's role as wife and mother. Other aspects of women's lives had to be fitted in around this responsibility.^53^ The model female figure was a full-time homemaker dependent upon her breadwinner husband, who had two, three or four children, and lived within a nuclear family.^54^ With their low rates of employment and high marriage rates Jewish women therefore fulfilled the 1950s ideal.^55^ However, refugee women often did work which marked them out from the established Jewish community.

Both before and after the war the easiest way for refugee women to come to Britain was as a domestic servant. Young single women predominated but married women with children also entered this way. Not all women who came on the domestic service schemes intended to remain in such work although it could be difficult to leave. While some women had positive stories to tell, it was often a difficult situation for women who came from middle-class families, who had often employed servants themselves, to adjust to being domestic servants in other families' homes.^56^ Rosa Yudt was born in Berlin to Polish parents. She came to England in 1939 as a domestic servant because it was ‘the only opportunity that I could get a visa for'.^57^ On arrival she went to live with her uncle in Bournemouth who ran a boarding house. When asked what sort of welcome she received she replied:

They not only didn't welcome they exploited me … my parents wanted me to go there to the uncle you know. They thought I'd be better off. I would've been better off not to go there.^58^

Ilse Sinclair was born in 1921 in Hamburg and also came to England 1939 as domestic. She also found that people were ready to take advantage of her situation. Her first interview was with ‘a very unpleasant Jewish family in the East End of London, and they offered me a very, very low salary, and I realised that they thought they'd have a bargain on their hands'.^59^ The next job Ilse went for, also with a Jewish family, was no better, ‘the attitude of the aunt was, “You're a little German Jewish refugee, and you just do as you're told and be grateful for it”'.^60^ Kushner found that many refugees remembered that their worst treatment was in Jewish homes, which he believes resulted from the marginal middle-class status of British Jewry.^61^ Anna Smith had come to England from Czechoslovakia on the Kindertransport in July 1939. Her parents, who worked in the family paper manufacturing business, were supposed to join her on domestic service permits but war broke out before they could leave:

Although my parents weren't servant types everyone who came as a refugee had to be a servant-type person. And my adoptive mother or foster mother as she then was or after, always made it absolutely clear to me in words, not just by implication that I was the child of servants and that I didn't deserve anything more, that I was lucky to be alive.^62^

Domestic service was also the usual route into England after the war. Rena Zabielak, who came to England in 1946 to marry her husband, explained that it was very difficult for her to get into Britain. She could only come as a domestic, although she did not actually intend to work in service. This was problematic as if women broke the terms of their permits they were under threat of removal. Rena recalled that she had ‘trouble’ when the authorities found out she was not actually working in domestic service after ‘somebody was here who didn't like us, and sent—wrote to the Home Office and the police came'. She added:

I took Henry [my son] to the Home Office and he said ‘Don't throw my mummy out, I am British’. Because he was born in England.^63^

Women who came as domestic servants could also try to find other types of work once in the country. Barbara Stimler was born in Poland in 1927 and came to England in 1946 to join family after surviving the concentration camps. She came as a domestic servant to work for her uncle. Her period of working for her uncle's family was not a happy time and Barbara found work as a seamstress in a factory.^64^ Margaret Augstein was born in 1913 in Czechoslovakia. She came to England in 1948 after marrying her second husband who had been in Britain during the war and sought British citizenship. Initially Margaret:

worked as a nurse in a private house. Not live-in, but as I had nursing experience from the war. It was to add to our income … [Then] I went to the hat classes and to English classes. And maybe in l950 I started making hats. And I must say was not unduly unsuccessful.^65^

However, the employment opportunities available to Jewish female refugees in the 1950s were limited. In part this was a legacy of their pre-war experiences. Interviewees who had been children or young women before and during the war had often had their education disrupted. Ilse Sinclair had come to England from Germany aged eighteen as a domestic servant, but had lost out on her last years of schooling due to the political situation. She had worked as a nanny before marriage by when asked whether she worked after having children she answered:

of course, I didn't have a proper profession, I mean … I hadn't really done anything. And my general knowledge was nil, my life experience was pretty big, but other than that, I mean, as far as scholarly knowledge goes, next to nothing.^66^

Even women who had qualifications and professional careers before coming to England could find it difficult to re-establish themselves in a new environment. Like many migrants they had to take jobs for which they were over-qualified. Gertrude Levi was born in 1924 in Hungary. She came to England in 1957 with her family. Married with one son, she was the family's main breadwinner as her husband, a musician, suffered from mental illness. She recalled:

I couldn't get a job as a teacher, because my qualifications [from Hungary] weren't recognised, and I found that I was doing, addressing envelopes at Advance Laundry, and working as a ledger clerk at a roof insulating manufacturer and getting any kind of, taking on any kind of work, whatever I could get.^67^

Lily Fischl, an artist, came to England in 1946 to join her children who had come on the Kindertransport. She had wanted to work as a miniature painter, but found she could not support herself. Instead she found herself working as a painter in a china repair shop. She remembered that it was ‘a sweat shop, it was really shocking … at the back, in the former kitchen of that little house, we worked, a few refugees [and] an invalid man who did the metal clamping'.^68^

Reflecting the negative attitudes towards married women's work in the Jewish community, and indeed the ambivalence towards it within wider British society, some interviewees presented work as a challenge to family life. Barbara Stimler worked as a tailoress before and after marriage. Her husband ‘opened his own factory and I was helping him, dragging Harvey [their son] with me every day then I put him in a nursery but he didn't like it he made himself ill and it was very difficult for me'.^69^ Similarly Rosa Yudt, who ran a dry cleaning business with her husband said:

I didn't have enough time to give to them [I was] trying to make a living really, so I was really sort of in half, you know, I felt I should be more with them … and it was very, very difficult.^70^

Both Barbara and Rosa justified their need to work as presenting it as enabling their families to achieve the ultimate goal of owning their own homes. The hardships they and their children endured were worthwhile as in the long term it helped them achieve the ideal of domesticity that they sought. Barbara explained:

we were thinking of the future, to secure ourselves. And coming from [a] home where my mother was very tidy and had always a lovely, lovely home, I was putting everything in my home.^71^

When asked in her interview whether she had ‘an idea in your mind of how you wanted their [her children's] home to be?’ Rosa answered:

A warm, a warm, warm home, you know. Yes, yes. They always had plenty of friends they could bring in, they used to bring in all the neighbourhood kids, and were very happy.^72^

Creating a home was an important theme among the interviewees. Gertrude Levi talked of trying to establish a home in the family's first flat of their own in the late 1950s. She said: ‘it took quite a long time, until I got some decency into it, and put it into, into a shape that I wanted it to be in.’^73^ Edith Birkin moved into the first home of her own with the man who later became her husband in 1961. She said:

And it was lovely. We bought some furniture and did it up, for the first time I had a home of my own. My own furniture and my own independence. I didn't have to––share with anybody, you know, we all shared one bathroom, the whole houseful, you know, and nobody could tell me what I was allowed to cook and what I wasn't allowed to cook, because it smelt.^74^

However, as their accounts indicate, the interviewees, like other post-war migrant groups,^75^ found they were often at the bottom of the housing ladder and had to live in the areas from which British Jews were moving out.^76^ Ruth Foster and her husband lived in Tottenham throughout the 1950s. She recalled that while most people ‘moved from the East End to North West and North London’, she and her husband, a doctor:

did the opposite move. Because my husband had just enough money to put a deposit, £250 saved by then … and he paid a deposit on a house in Tottenham, in Broad Lane. And [that's] where he squatted. I call it squatting. He just put a name-plate up. And I decorated the house, it was a real hovel.^77^

After marrying her husband, also a Polish Jewish immigrant, in the late 1940s, Barbara Stimler rented a flat from her uncle:

it was the horrible place where I went to collect the rent with my cousin. My God if I had known at that time that I'm going to live there I certainly would've went back home, even to Poland, never mind to Germany.^78^

She continued: ‘So we were living in that Cable Street for a year … My uncle asked me thirteen shillings a week for the rent for this place, which I found out afterwards the previous tenants only paid ten and six.’^79^ After the couple had been living in the flat a year:

the police just opened the door and … he said, ‘I am terribly sorry but you should keep your doors closed, you know what kind of people are living here?’ I said ‘I don't’. He said, ‘They are gangsters and prostitutes. I went downstairs in the basement and a man was ehm sleeping there with a few women, so you should, must keep your door closed, locked.’^80^

As noted above, Barbara and her husband eventually escaped their poor housing by saving enough to buy a house of their own. Gertrude Levi and her family came to England from Hungary via Israel and South Africa in 1957. She said the family shared one room which was:

absolutely abysmal, awful, dirty place, where we had to share the bathroom with, I don't know how many people, about, about twenty-two people, and I had to cook on a one-ring, on which, which was on the floor, and really was, I mean, absolutely awful.^81^

Describing her first house in London in the late 1940s Margaret Augstein recalled that:

The living conditions were—very, very bad. You know, I never saw anything like it. There was a carpet which I washed every—every so often, every week at least I washed it. It was always the same blackness. The kitchen had no window, it was a little kitchenette. And, you know, I scrubbed that cooker in and out, it never got clean. I lived there for one and a half years, it never got clean.^82^

While having a home of your own was a common desire for many women in 1950s Britain,^83^ refugee women were also trying to recreate a family life that had been taken from them. Recalling her loneliness during the 1950s Edith Birkin explained:

Although I lived with my friend, and I had friends, I wanted something more, I wanted … to be involved and have a family and, have a good husband and have children and, you know, to re-create what I'd lost.^84^

Similarly when Rosa Yudt was asked during her interview ‘Was it very important to you to have children, make a family?’ she replied, ‘Oh yes … Very important, yes'. However, she also expressed her concerns that because she worked she did not meet the ideal of good motherhood she aspired to.^85^ Freda Wineman had come to England in 1950 to marry her husband, a distant relation she had met on holiday in England. She was left a widow to two young children when he died two years later. When asked during the interview why she did not get a job she explained:

I said, ‘I want them to grow up to be normal. To have a normal home life. Not to be deprived, not only of a father, but to be also deprived of a mother.’ There was no real family background here for us, for them. So they were brought up, but their mother was always at home. I helped them with their homework. I was there when they needed me.^86^

However, as Freda intimated in her account, building their new families in England could also remind interviewees of their lack of family. Claudette Kennedy had come to England in 1950 to marry her British husband. She recalled, ‘it was very hard on me coming from France where I was surrounded with friends and affection'. She felt very isolated.^87^ Edith Birkin explained that she was left with ‘this sort of loneliness in me’. While she had friends who were helpful ‘nothing could substitute what I lost'.^88^ Wlodja Robertson also recalled her mixed feelings when she was bringing up her young children in the 1950s:

I had no experience really, of normal family life, but there were a lot of young women with first babies, and they were all very friendly and helpful to me … And my sister had, by that time, emigrated with her husband to America, so I did feel lonely, and it was lucky that there were all these friendly people.^89^

## Identity

These feelings of loss and isolation were common. Phyllis Lassner suggests that Jewish refugees ‘occupied a precariously in-between space in British society, always learning to be innocuous but recognized nonetheless as different in ways that could never fit any British cultural setting or cultural category'.^90^ In order to relieve the ‘frustration that could come from lack of comprehension or sheer disbelief’ about the holocaust, Kushner suggests that survivors found that mutual support ‘became important from the start of their lives in Britain’.^91^ For the refugee and survivor women in this sample, the only time that many did not feel a sense of being out of place was when they were amongst themselves. For example when asked whether she felt an affinity to people who had been in the camps Claudette Kennedy explained that she thought there was:

a kind of approach to life, I suppose, which is common and which makes communication easier. I think I communicate with ex-prisoners whatever their background is. Better than with anybody else.^92^

In a similar fashion to other migrant groups in the post-war decades,^93^ several interviewees recalled how they socialised almost exclusively with other people who had a similar background to themselves. For example, describing life in the 1940s and 1950s, Ilse Sinclair said:

I mixed with a lot of Jews, and we belonged to an International Club in Guildford. It was full of Jews, full of refugees. They were mostly refugees from Germany, Czechoslovakia, Austria, and places like that.^94^

Remembering living in London in the late 1940s and early 1950s Wlodja Robertson, said, ‘I think we were slightly oblivious to the local people, and were interested to meet people who had also come from Poland here'.^95^ Rosa Yudt also found that she felt closest to other refugees: ‘we found we'd got something in common, you know, and sort of a common bond between us.'^96^ As a girl growing up in the 1950s Caroline Blank recalled that her parents’ circle of friends ‘was virtually exclusively other survivors and refugees'.^97^ Moreover Caroline herself found that she found it easiest to get on with the children of other survivors.^98^

However, another strategy that these Jewish interviewees recalled employing in order to fit in was trying to hide their identity. None of the interviewees in this sample went as far as denying their Jewish background although they did know family members who had done so.^99^ Several of the interviewees said they were reticent about discussing their Jewishness. Caroline Blank, who was born in London in 1948, explained:

when I was growing up that I tended not to volunteer that I was Jewish. I never hid it. But I didn't  …  I didn't come out and volunteer it unless I had to.^100^

Jennifer Wingate also grew up in the 1950s, she was born in London in 1944 to parents of an East European background. Whilst interviewing her mother, Mary Roslyn, she recalled:

I remember when I was a girl, a young girl, going out dating, not necessarily dating, just going out with friends, mixed friends, you would say to me, ‘There is no need to tell people you are Jewish.’ Because you were trying to protect me.^101^

As noted above, Anna Smith had a troubled childhood, coming to England from Czechoslovakia as a refugee to live with a foster mother with whom she did not have a good relationship. Her negative experiences encouraged her to reject her Jewish background. Indeed her difficulty in reconciling her English and Czech identities was a recurrent theme in her narrative.^102^ She said she was pleased after the war when her foster family adopted her because she was ‘very embarrassed about being Jewish by then and being adopted allowed me to change my name'.^103^

In contrast many interviewees said that there Jewish identity was essential to them. Rosa Yudt had come to England in 1939 as a domestic servant but during the war she joined the ATS. She remembered it is being a very difficult time because she felt she had to hide the fact she was from Germany and pretend she was from Poland.^104^ She said she ‘felt a misfit’.^105^ However, Rosa took consolation in that she was able to maintain her Jewish identity: ‘I was always proud of that you know.'^106^ Xenia Kahan was born in 1907 in Russia. She arrived in England as a child in 1919 with family after fleeing from the Revolution. Discussing whether she felt Russian or British Xenia answered, ‘I am Jewish and nothing else'.^107^ Gertrud Levy was born a generation later in 1924 in Hungary. She also had a very different migration history. After surviving Auschwitz and Hessisch-Lichtenau she had lived in France, South Africa and Israel before coming to England in in 1957 with her husband and young son. However, similarly to Xenia Kahan, when asked whether she felt Hungarian or British she replied, ‘I'm *only* Jewish'.^108^ Interestingly discussing her feelings about her Jewishness Wlodja Robertson, who came to England from Poland in 1946, felt her Jewishness was an external rather than internal identity:^109^ ‘I've been made to know I'm Jewish by everybody round me for so long, that … I have no doubts about what I am!'^110^ Their feeling of not being fully accepted in British society perhaps also influenced their ambivalent attitudes towards living in Britain. Many interviewees said they did not feel British, even if they were glad they had made Britain their home.^111^ For example Rosa Yudt summed up her mixed feelings about her adopted country: ‘I like it, it's a very pleasant country to live in, very very nice. Lovely life, but I don't feel one hundred percent involved.’^112^

## Conclusion

This article has addressed questions of belonging and ‘unbelonging’ in oral history narratives of Jewish refugees and survivors about their lives in 1950s Britain. Through looking at their accounts of arrival, work and home, and identity it has shown while there were similarities in between their lives and British Jews, other migrant groups and women in 1950s Britain more generally, their experiences were also distinct from each of these groups, for example in their desire to recreate the family life that had been taken from them. Antisemitism, and more specifically the fear of antisemitism, made it difficult for women to feel they were part of the British nation. Their accounts indicate that not only did they find it hard to feel at home in Britain, but also amongst the British Jewish community, and they believed they faced hostility at all levels. Indeed some had painful stories of rejection at the hands of their own family. They employed a number of strategies to try and feel more ‘in place’, such as concealing their identity so they could ‘fit in’, taking pride and comfort in their Jewish identity, or surrounding themselves with other people like themselves. Through depicting their uneasy reception of these refugee and survivor women, the article also reveals something of the nature of British Jewry and wider British society. However, the article has also demonstrated the need for more research in this understudied area particularly on the relationship between refugees and survivors, British Jews and wider society in post-war Britain.

## Notes

1 Wendy Webster (1998) *Imagining Home: gender, ‘race' and national identity, 1945–1964* (London: University College London Press), p. 40.

2 Ibid. pp. 41–43.

3 Tony Kushner (1991) An Alien Occupation: Jewish refugees and domestic service in Britain, 1933–1948, in Werner E. Mosse & Julius Carlebach (Eds) *Second Chance: two centuries of German-speaking Jews in the United Kingdom* (Tübingen: J. C. B. Mohr [Paul Siebeck]) pp. 553–587, p. 553.

4 Lara Marks (1992) Carers and Servers of the Jewish Community: the marginalized heritage of Jewish women in Britain, in Tony Kushner (Ed.) *The Jewish Heritage in British History: Englishness and Jewishness* (London: Cass), pp. 106–127, p. 108.

5 Marks, ‘Carers and Servers’, p. 107; Rickie Burman (1990) Jewish Women and the Household Economy in Manchester, c. 1890–1920, in David Cesarani (Ed.) *The Making of Modern Anglo-Jewry* (Oxford: Basil Blackwell), pp. 55–75, p. 55.

6 Paula E. Hyman (2002) Gender and the Shaping of Modern Jewish Identities, *Jewish Social Studies*, 8, pp. 153–161, p. 154. Rickie Burman (1982) The Jewish Woman as Breadwinner: the changing value of women's work in a Manchester immigrant community, *Oral History*, 10, pp. 27–39; Burman, ‘Jewish Women and the Household Economy’; Lara Marks (1990) ‘Dear Old Mother Levy's’: the Jewish maternity home and sick room helps society 1895–1939, *Social History of Medicine*, 3, pp. 61–88; ‘Carers and Servers’; (1992) The Luckless Waifs and Strays of Humanity: Irish and Jewish immigrant unwed mothers in London, 1870–1939, *Twentieth Century British History*, 3, pp. 113–137; (1994) *Model Mothers: Jewish mothers and maternity provision in east London 1870–1939* (Oxford: Clarendon Press); (1996) *Metropolitan Maternity: maternal and infant welfare services in early twentieth century London* (Amsterdam: Rodopoi); Susan Tananbaum (1993) Making Good Little English Children: infant welfare and Anglicization among Jewish immigrants in London, 1880–1939, *Immigrants Minorities*, 12, pp. 176–199; (1994) Biology and Community: the duality of Jewish mothering in East London, 1880–1939, in E.N. Glenn, G. Chung & L. Rennie Forcey (Eds) *Mothering: ideology, experience and agency* (London: Routledge), pp. 311–332; (1997) Philanthropy and Identity: gender and ethnicity in London, *Journal of Social History*, 30, pp. 937–961; (2010) Democratizing British-Jewish Philanthropy:the Union of Jewish Women (1902–1930), *Nashim*, 20, pp. 57–79; (2014) *Jewish* *Immigrants in London, 1880–1939* (London: Pickering & Chatto); Anne Summers (2012) Gender, Religion and an Immigrant Minority: Jewish women and the suffrage movement in Britain c. 1900–1920,*Women's History Review*, 21, pp. 399–418; (2014) False Start or Brave Beginning? The Society of Jews and Christians, 1924–1944, *Journal of Ecclesiastical History*, 65, pp. 827–851.

7 Tony Kushner (1993) Sex and Semitism: Jewish women in Britain in war and peace, in P. Panikos (Ed.) *Minorities in Wartime: national and racial groupings in Europe, North America and Australia during the two World Wars* (Oxford: Berg), pp. 118–132, p. 119.

8 Tony Kushner (1988) Asylum or Servitude? Refugee domestics in Britain, 1933–1945, *Bulletin of the Society for the Study of Labour History*, 53, pp. 19–27; (1989) Politics and Race, Gender and Class: refugees, fascists and domestic service in Britain, 1933–40, *Immigrants Minorities*, 8, pp. 49–58; ‘An Alien Occupation’; ‘Sex and Semitism’. There have also been studies of the reaction of British women to these refugees. Helen Jones has studied the role of British women in welcoming refugees and challenging antisemitism in the 1930s and 1940s: Helen Jones (2012) National, Community and Personal Priorities: British women's responses to refugees from the Nazis, from the mid-1930s to early 1940s, *Women's History Review*, 21, pp. 121–151. Julie Gottlieb has analysed the involvement of women within the British fascist movement during this period: Julie Gottlieb (2002) ‘Motherly Hate': gendering anti-Semitism in the British Union of Fascists, *Gender and History*, 14, pp. 294–320.

9 Kushner, ‘Sex and Semitism’, 149.

10 Joanne Weiner Rudof (2006) Research Use of Holocaust Testimonies, *Poetics Today*, 27, pp. 451–461, p. 454.

11 Ruth Linden (1996) Troubling Categories I Can't Think Without: reflections on women in the Holocaust, *Contemporary Jewry*, 17, pp. 18–33; Susan Rubin Suleiman (2002) The 1.5 Generation: thinking about child survivors and the Holocaust, *American Imago*, 59, pp. 277–295, pp. 286–287.

12 April Gallwey (2012) The Rewards of Using Archived Oral Histories in Research: the case of the Millennium Memory Bank, *Oral History*, 41, pp. 37–50; Joanna Bornat, Parvati Raghuram & Leroi Henry (2012) Revisiting the Archives—Opportunities and Challenges: a case study from the history of geriatric medicine, *Sociological Research Online*, 17, www.socresonline.org.uk/17/2/11.html; Ronald Grele (1987) On Using Oral History Collections: an introduction, *The Journal of American History,* 74, pp. 570–578; P. Jackson, G. Smith & S. E. Olive (2007) *Families Remembering Food: reusing secondary data*, www.sheffield.ac.uk/polopoly_fs/1.145076!/file/FRF-reuse-paper-WP-.pdf; N. Moore (2007) (Re)Using Qualitative Data?, *Sociological Research Online*, 12, www.socresonline.org.uk/12/3/1.html.

13 Jackson, Smith & Olive, *Families Remembering Food*, p. 20.

14 Tony Kushner (2006) Holocaust Testimony, Ethics, and the Problem of Representation, Poetics Today, 27, pp. 275–295, p. 275.

15 Rachel Einwohner (2011) Ethical Considerations on the Use of Archived Testimonies in Holocaust Research: beyond the IRB exemption, *Qualitative Sociology*, 34, pp. 415–430, p. 424.

16 J. L. Jacobs (2004) Women, Genocide, and Memory: the ethics of feminist ethnography in Holocaust research, *Gender and Society*, 18, pp. 223–238, p. 227.

17 Natasha Burchardt has noted that most scholarship on holocaust survivors and their families has focused on those living in Israel and the United States: Natasha Burchardt (1995) Transgenerational Transmission in the Families of Holocaust Survivors in England, in Daniel Bartaux & Paul Thompsn (Eds) *Between Generations: family models, myths and memories* (Oxford: Oxford University Press), pp. 121–137, pp. 121–122.

18 Bronwen Walter (2010) Irish/Jewish Diasporic Intersections in the East End of London: paradoxes and shared locations, in M. Prum (Ed.) *La Place de* *l'Autre* (Paris: L'Harmattan Press), pp. 53–67, p. 66.

19 Tony Kushner (1994) *The Holocaust and the Liberal Imagination: a social and cultural history* (Oxford: Blackwell), p. 273; Bernard Wasserstein (1979) *Britain and the Jews of Europe 1939–1945*, (London: Institute of Jewish Affairs), pp. 349–356; Louise London (2000) *Whitehall and the Jews, 1933–1948: British immigration policy, Jewish refugees and the holocaust* (Cambridge: Cambridge University Press), pp. 1–2.

20 London, *Whitehall and the Jews*, pp. 11–12.

21 Jillian Davidson, German-Jewish Women, in Mosse & Carlebach, *Second Chance*, p. 539.

22 Phyllis Lassner (2008) *Anglo-Jewish Women Writing the Holocaust: displaced witnesses* (Basingstoke: Palgrave Macmillan), p. 6.

23 Linda McDowell (2004) Narratives of Family, Community and Waged Work: Latvian European volunteer worker women in post-war Britain, *Women's History Review*, 13, pp. 23–56, p. 24; Kushner, *Holocaust and the Liberal Imagination*, p. 235; Kushner, ‘An Alien Occupation’, p. 575

24 Todd Endelman (2002) *The Jews of Britain, 1656 to 2000* (Berkeley: University of California Press), pp. 231–232.

25 Webster, *Imaging Home*, pp. 41–44.

26 British Library, Living Memory of the Jewish Community, C410/025, p. 68. All following citations of this sort are from the Living Memory of the Jewish Community collection (C410) held at the British Library.

27 C410/030, p. 170.

28 Ibid. p. 188.

29 Ibid. p. 169.

30 C410/012, pp. 41–42.

31 C410/004, p. 97

32 C410/004, p. 99.

33 The Act offered British citizenship to over 200,000 displaced Polish troops on British soil who had fought against Nazi Germany and opposed the Soviet takeover of their homeland.

34 C410/102, p. 53.

35 C410/051, p 42.

36 C410/024, p. 11.

37 Ibid*.* p. 10.

38 C410/007, pp. 27–28. A pseudonym has been used as the interviewee wished to preserve her anonymity.

39 C410/027, pp. 52–53.

40 C410/004, p. 98.

41 C410/013, p. 91.

42 C410/017, p. 30.

43 C410/041, p. 54.

44 Dolly Smith Wilson (2006) A New Look at the Affluent Worker: the good working mother in post-war Britain, *Twentieth Century British History*, 17, pp. 206–229, p. 225.

45 Webster, *Imagining Home*, p. 127.

46 Hannah Neustatter (1955) Demographic and Other Statistical Aspects of Anglo-Jewry, in Maurice Freedman (Ed.) *A Minority in Britain: social studies of the Anglo-Jewish community* (London: Valentine Mitchell), pp. 55–133, pp. 124–126. British Jewry became overwhelmingly middle-class and by the early 1960s over 40% of British Jewry was located in the upper two social classes.

47 Kushner, ‘Sex and Semitism’, pp. 138–139; Endelman, *The Jews of Britain*, p. 240; Geoffrey Alderman (2003) British Jewry: the disintegration of a community, in Sol Encel & Leslie Stein (Eds) *Continuity, Commitment, and Survival: Jewish communities in the diaspora* (Westport: Praeger), p. 50.

48 Neustatter, ‘Statistical Aspects’, p. 126.

49 Ibid. p. 91.

50 Eileen Janes Yeo (2010) Gender and Homeland in the Irish and Jewish Diasporas, 1850–1930, in Marlou Schrover & Eileen Janes Yeo, *Gender, Migration and the Public Sphere* (New York: Routledge), pp. 14–37.

51 Shoshana Ringel (2007) Identity and Gender Roles of Orthodox Jewish Women, *Smith College Studies in Social Work*, 77, pp. 25–44.

52 Elizabeth Wilson (1997) *Women and the Welfare State* (London: Tavistock), p. 35.

53 Birmingham Feminist History Group (1979) Feminism as Femininity in the Nineteen-Fifties?, *Feminist Review*, 3, pp. 48–65, p. 50.

54 Angela Davis (2012), *Modern Motherhood: women and family in England c. 1945–2000* (Manchester: Manchester University Press), p. 177.

55 Lara Marks has demonstrated how Jewish women were seen as ‘model’ mothers in the first half century and were praised for their homemaking: Marks, *Model Mothers*. 

56 Tony Kushner has discussed the employment of Jewish refugee women in domestic service and the difficulties they faced at length: Kushner, ‘Asylum or Servitude’; ‘Politics and Race'; ‘An Alien Occupation’; ‘Sex and Semitism’.

57 C410/006, p. 30.

58 Ibid. pp. 34–35.

59 C410/011, p. 46.

60 Ibid. p. 46.

61 Kushner, ‘An Alien Occupation’, p. 567.

62 C410/007, p. 24.

63 C410/041, p. 52

64 C410/004, pp. 98–99.

65 C410/012, p. 31.

66 C410/011, p. 58.

67 C410/066, p. 199.

68 C410/095, p. 136.

69 C410/004, p. 115.

70 C410/006, pp. 57.

71 C410/004, p. 124.

72 C410/006, p. 58.

73 C410/005, p. 234.

74 C410/030, p. 189.

75 Webster, *Imagining Home*, p. 173.

76 The blitz and its aftermath encouraged Jews to move out of the East End to suburbs such as Hackney (which had the largest and densest Jewish population in the 1950s), Golders Green, Edgware, Finchley and Hendon. The large provincial communities—Manchester, Leeds, Glasgow—also became overwhelmingly suburban in the second half of the century: Endelman, *The Jews of Britain*, p. 230.

77 C410/102, p. 55.

78 C410/004, p. 111.

79 Ibid. pp. 112–113

80 Ibid. pp. 113–114.

81 C410/055, p. 234.

82 C410/012, pp. 41–42.

83 Davis, *Modern Motherhood*, pp. 23–25.

84 C410/030. p. 187.

85 C410/006, p. 57.

86 C410/013, p. 101.

87 C410/027, p. 53.

88 C410/030, p. 177.

89 C410/025, p. 77.

90 Lassner, *Anglo-Jewish Women*, p. 12.

91 Kushner, *Holocaust and the Liberal Imagination*, pp. 228–229. Kushner claims that while the atrocity material of 1945 made an impression on the British public many people wanted to forget as quickly as possible. While largely supporting Kushner's conclusions, David Cesarani believes that throughout the 1950s there was ‘a dim awareness that something terrible had happened in Europe under the Nazis as a result of which six million Jews perished and the State of Israel came into existence'. The ‘silence’ on the Holocaust that existed in Britain in the 1950s was a result of ‘partial knowledge rather than complete ignorance': Kushner, *Holocaust and the Liberal Imagination*; David Cesarani (2010) How Post-war Britain Reflected on the Nazi Persecution and Mass Murder of Europe's Jews: a reassessment of early responses, *Jewish Culture and History*, 12, pp. 95–130, p. 124.

92 C410/027, p. 49.

93 Webster, *Imagining Home*, p. 180.

94 C410/011, p. 52.

95 C410/025, p. 71.

96 C410/006, p. 56.

97 C410/123, p. 17.

98 Ibid. p. 53. Natasha Burchardt found children of survivors were frequently drawn to other children of survivors': Burchardt, ‘Transgenerational Transmission’, pp. 130–131.

99 C410/185, p. 137; C410/095, p. 134; C410/012, p. 57.

100 C410/123, p. 21.

101 C410/185, p. 137.

102 The struggle over identity faced by child survivors and refugees has been addressed by Susan Rubin Suleiman: Suleiman ‘The 1.5 Generation’. The particular problems of child children from the Kindertransport has also been discussed by Phyllis Lassner: Lassner, *Anglo-Jewish Women*.

103 C410/007, p. 37. A number of the women recalled that their families had changed their Central and Eastern European surnames to something more English sounding: C410/185, p. 13; C410/004, p. 109; C410/011, p. 58; C410/102, p. 53.

104 As the daughter of Polish immigrants to Germany she had a Polish passport.

105 C410/006, p. 40.

106 Ibid.

107 C410/075, pp. 69–70.

108 C410/066, p. 238.

109 Manuela Barreto and Naomi Ellemers (2003) The Effects of Being Categorised: the interplay between internal and external social identities, *European Review of Social Psychology*, 14, pp. 139–170.

110 C410/025, p. 81.

111 C410/011, p. 83; C410/095, p. 155; C410/012, p. 53; C410/017, p. 29; C410/075, pp. 69–70.

112 C410/006, p. 73.

